# A systematic review with attempted network meta-analysis of asthma therapy recommended for five to eighteen year olds in GINA steps three and four

**DOI:** 10.1186/1471-2466-12-63

**Published:** 2012-10-15

**Authors:** Lonneke B van der Mark, PH Edo Lyklema, Ronald B Geskus, Jacob Mohrs, Patrick JE Bindels, Wim MC van Aalderen, Gerben ter Riet

**Affiliations:** 1 Division of Clinical Methods & Public Health, Department of General Practice, Academic Medical Center-University of Amsterdam, P.O. Box 22700, Amsterdam, 1100 DD, The Netherlands; 2 Department of Clinical Epidemiology, Biostatistics, and Bioinformatics, Academic Medical Center, Amsterdam, The Netherlands; 3 Department of General Practice, Erasmus Medical Center, Rotterdam, The Netherlands; 4 Department of Paediatric Respiratory Medicine and Allergy, Emma Children’s Hospital – Academic Medical Center, Amsterdam, The Netherlands

**Keywords:** Adolescent, Asthma, Child, Meta-analysis, Randomized controlled trial

## Abstract

**Background:**

The recommendations for the treatment of moderate persistent asthma in the Global Initiative for Asthma (GINA) guidelines for paediatric asthma are mainly based on scientific evidence extrapolated from studies in adults or on consensus. Furthermore, clinical decision-making would benefit from formal ranking of treatments in terms of effectiveness.

Our objective is to assess all randomized trial-based evidence specifically pertaining to 5-18 year olds with moderate persistent asthma. Rank the different drug treatments of GINA guideline steps 3&4 in terms of effectiveness.

**Methods:**

Systematic review with network meta-analysis. After a comprehensive search in Central, Medline, Embase, CINAHL and the WHO search portal two reviewers selected RCTs performed in 4,129 children from 5-18 year old, with moderate persistent asthma comparing any GINA step 3&4 medication options. Further quality was assessed according the Cochrane Collaboration’s tool and data-extracted included papers and built a network of the trials. Attempt at ranking treatments with formal statistical methods employing direct and indirect (e.g. through placebo) connections between all treatments.

**Results:**

8,175 references were screened; 23 randomized trials (RCT), comparing head-to-head (n=17) or against placebo (n=10), met the inclusion criteria. Except for theophylline as add-on therapy in step 4, a closed network allowed all comparisons to be made, either directly or indirectly. Huge variation in, and incomplete reporting of, outcome measurements across RCTs precluded assessment of relative efficacies.

**Conclusion:**

Evidence-based ranking of effectiveness of drug treatments in GINA steps 3&4 is not possible yet. Existing initiatives for harmonization of outcome measurements in asthma trials need urgent implementation.

## Background

Clinical guidelines contain systematically developed statements to help practitioners make optimal healthcare decisions [1]. The Global Initiative for Asthma (GINA) guideline is a major step forward in achieving best possible asthma control [2]. The GINA guideline uses symptoms, exacerbations, airflow limitation, and lung function variability to categorize asthma severity into *intermittent, mild persistent, moderate persistent* or *severe persistent*. GINA suggests that 5 to 18 year-olds, whose symptoms are insufficiently controlled after three months of treatment at a particular GINA step, move up a step (see Table 1).

**Table 1:** GINA recommended treatment steps for 5 to 18 year olds

**Step 1**	**Step 2**	**Step 3 (Select one)**	**Step 4 (Add one or more)**
SABA	Low dose ICS	A. Medium-or high dose ICS	A. Medium-or high dose ICS + LABA
		B. Low dose ICS + LABA	B. LTRA
		C. Low dose ICS + LTRA	C. Theophylline
		D. Low dose ICS + Theophylline	

SABA = rapidly acting ß_2_-agonists; ICS = Inhaled Corticosteroids; LABA = Long–acting β_2_-adrenoceptor agonists; LTRA = Leukotriene modifier.

There is level A evidence (see glossary) on the effectiveness of short acting ß_2_-agonists (SABA; step 1) and adding a low dose inhaled glucocorticosteroid (ICS) (step 2) in children with mild asthma [2]. However, although the level of evidence for GINA step 3&4 recommendations for children older than 5 years is deemed A to B, level A evidence to guide step-up therapy is lacking for this age group. Scrutiny of the randomized trials (RCTs) underlying the guideline, reveals that some are outdated, because children used daily oral prednisone (see for example [3,4]), or compare step 2 with step 3 (see for example [5]). This leaves only five RCTs comparing treatments of step 3&4 for this age group [6-10].

Network meta-analysis (NMA), also known as indirect comparisons, exploits the mathematical property that (A – B) – (A – C) = A – B – A + C = C – B. It enables one to formally compare drugs B and C although these were never compared head-to-head [11-13]. NMA has major advantages over classic meta-analysis; it formally ranks treatment effects in case more than two treatments are involved; it circumvents the usual overrepresentation of drug comparisons to placebo, which may not always be the most informative for practising physicians [14]. We set out, using NMA methodology, to compare GINA step 3&4 drug treatment efficacies in 5 to 18 year-old children/adolescents with moderate persistent asthma.

## Methods

### Search strategy

A trained clinical librarian performed a comprehensive literature search for relevant RCTs in the Cochrane Central Register of Controlled Trials (Central), Medline (Pubmed), Embase, CINAHL and ongoing trial registers registered on WHO Search Portal [15], published until 4 February 2010 (For search details, see Additional file 1). In addition, two reviewers (LvdM, PhEL) scrutinized reference lists of included articles, the GINA-guideline and relevant systematic reviews.

### Inclusion and exclusion criteria

We included RCTs conducted in participants aged 5 to 18 years with persistent-moderate asthma and comparing any GINA step 3&4 medication options (see Additional file 2) to each other or against placebo, with a follow-up duration of at least four weeks after start of the intervention. There were no language restrictions. Acceptable outcome measurements were: spirometry (forced expiratory volume in 1 second (FEV_1_)_,_ forced vital capacity (FVC), FEV_1_/FVC ratio, forced expiratory flow 25%-75% (FEF_25-75_), peak expiratory flow (PEF)), methacholine challenge test (PC_20_-FEV_1_), fractional exhaled Nitric Oxide (FeNO), asthma symptom score, use of ß_2_-agonists as breakthrough medication, and quality of life.

If results did not pertain to the 5 to 18 years age category, the trial was excluded with one exception: RCTs including 4-year olds were included if mean or median age was between 5 to 18 years. Studies were excluded if they compared add-on medication to a non-standardised dose of ICS. Cross-over studies not reporting on treatment effects for each separate treatment period were also excluded since carry-over effects cannot be excluded and are extremely difficult to handle [16].

### Selection

Two reviewers (LvdM, PhEL) independently assessed titles and abstracts of all identified citations against the inclusion criteria. Any disagreements were resolved by consensus; in case of doubt references were included. LvdM and PhEL evaluated in full text all papers thus selected against the inclusion criteria.

### Data extraction

LvdM and PhEL extracted, not in duplicate, data on author, source and year of publication, language, study design, interventions (medication, way of administration, dose, and frequency), population summary characteristics (me(di)an age, asthma severity) and study outcomes. If possible, we calculated the mean change from baseline to endpoint for each trial arm. To facilitate meta-analysis, we contacted authors and sponsors of included studies for additional information such as outcomes expressed on other scales, (mean) patient characteristics such as height, and statistics such as standard errors if needed. We asked for separate data (summaries) of participants in the 5 to 18 years range if the trial had combined this group with younger or older participants.

### Quality assessment

Methodological quality of all included trials was assessed on 9 items [17,18] (see Table 2). The risk of bias scale was developed using the Cochrane Collaboration’s tool for assessing risk of bias [17]. All items were scored as “yes” for low, “no” for high, and “?” for uncertain risk of bias, respectively.

**Table 2:** Risk of bias in included trials

**Study ID[ref]**	**1 ** [**19**]	**2 ** [**20**]	**3 ** [**21**]	**4 ** [**22**]	**5 ** [**23**]	**6 ** [**24**]	**7 ** [**25**]	**8 ** [**26**]	**9 ** [**7**]	**10 ** [**9**]	**11 ** [**27**]	**12 ** [**28**]	**13 ** [**29**]	**14 ** [**30**]	**15 ** [**31**]	**16 ** [**8**]	**17 ** [**32**]	**18 ** [**33**]	**19 ** [**6**]	**20 ** [**34**]	**21 ** [**10**]	**22 ** [**35**]	**23 ** [**36**]	**Y**	**N**	**?**
**Sequence generation**	**?**	**?**	**?**	**Y**	**?**	**Y**	**?**	**?**	**Y**	**?**	**?**	**?**	**Y**	**Y**	**?**	**?**	**Y**	**?**	**Y**	**?**	**Y**	**?**	**Y**	**9**	**0**	**14**
**Allocation concealment**	**?**	**?**	**Y**	**Y**	**?**	**?**	**?**	**?**	**Y**	**?**	**?**	**?**	**?**	**?**	**?**	**?**	**Y**	**?**	**?**	**?**	**?**	**?**	**Y**	**5**	**0**	**18**
**Appropriate blinding of participants**	**?**	**N**	**Y**	**N**	**?**	**?**	**?**	**Y**	**N**	**Y**	**?**	**?**	**?**	**?**	**?**	**?**	**Y**	**?**	**Y**	**?**	**?**	**N**	**Y**	**6**	**4**	**13**
**Appropriate blinding of outcome assessors**	**?**	**Y**	**Y**	**N**	**?**	**?**	**?**	**Y**	**?**	**Y**	**?**	**?**	**?**	**?**	**?**	**?**	**?**	**?**	**?**	**?**	**?**	**N**	**Y**	**5**	**2**	**16**
**Appropriate blinding of physician**	**?**	**?**	**Y**	**N**	**?**	**?**	**?**	**Y**	**?**	**Y**	**?**	**?**	**?**	**?**	**?**	**?**	**?**	**?**	**?**	**?**	**?**	**N**	**Y**	**4**	**2**	**17**
**Registration of loss to follow-up**	**Y**	**Y**	**?**	**?**	**Y**	**Y**	**?**	**?**	**Y**	**Y**	**Y**	**Y**	**Y**	**Y**	**Y**	**?**	**?**	**Y**	**?**	**Y**	**Y**	**Y**	**Y**	**16**	**0**	**7**
**Way missing values were dealt with**	**?**	**?**	**?**	**Y**	**?**	**?**	**?**	**?**	**Y**	**?**	**?**	**?**	**?**	**?**	**?**	**?**	**Y**	**Y**	**?**	**?**	**?**	**?**	**?**	**4**	**0**	**19**
**Compliance checked**	**Y**	**?**	**?**	**Y**	**Y**	**?**	**?**	**?**	**Y**	**?**	**?**	**Y**	**Y**	**Y**	**?**	**Y**	**?**	**Y**	**?**	**?**	**Y**	**?**	**Y**	**11**	**0**	**12**
**Selective reporting**	**?**	**?**	**?**	**?**	**?**	**N**	**?**	**?**	**?**	**?**	**?**	**?**	**?**	**?**	**?**	**?**	**?**	**?**	**?**	**?**	**?**	**?**	**?**	**0**	**1**	**22**
**Total times “Yes”**	**2**	**2**	**4**	**4**	**2**	**2**	**0**	**3**	**5**	**4**	**1**	**3**	**3**	**3**	**1**	**1**	**4**	**3**	**2**	**1**	**3**	**1**	**7**			
**Overall score Y/N/?**																								**60**	**9**	**138**

Each trial could score “yes” for a low, “no” for a high and “?” for an uncertain risk of bias, respectively.

The 9 items are based on a combination of the Cochrane approach to assess the risk of bias, combined with the validity checklist of Jadad et al. [17,18].

### Statistical analysis

Many network meta-analyses were based on dichotomous outcomes for each trial. In our study, outcomes were mostly continuous. To take lung function as an example, meta-analysis had been possible if, for each treatment arm, every publication had reported change in mean FEV_1_%_pred_ and its standard error after a suitable period of follow-up. Unfortunately, several trials only reported FEV_1_(l) therefore we did some efforts to salvage the problem by converting the FEV_1_(l)-value in FEV_1_%_pred_. Ideally, we would have had access to individual patient data (IPD) for each trial in the review. In our case we simulated IPD using the summary statistics reported. We simulated 1000 virtual children from a general population with age, height and sex distribution based on the available data on mean age, height and sex per trial arm. Next, we calculated a corresponding FEV_1_(l)-value per virtual child, using existing formula’s. In a final step, for each trial-arm, we tried to calculate a mean FEV_1_%_pred_ and a corresponding SD from the simulated data, to be used for meta-analysis. (For details on the statistical analysis see Additional file 3) [37-41].

We also considered the use of Z-scores. However, the SD was frequently missing and not provided after request. Furthermore, Z-scores can only be compared if the average of both outcomes (FEV_1_(l) and FEV_1_%_pred_) differ by a multiplicative factor, equal to the quotient of the standard errors. Since there were no studies that provided both FEV_1_(l) and FEV_1_%_pred_ and their respective standard errors, we could not check whether this property was approximately correct in our data and refrained from using this method.

## Results

### Studies and patients

**Figure 1:**
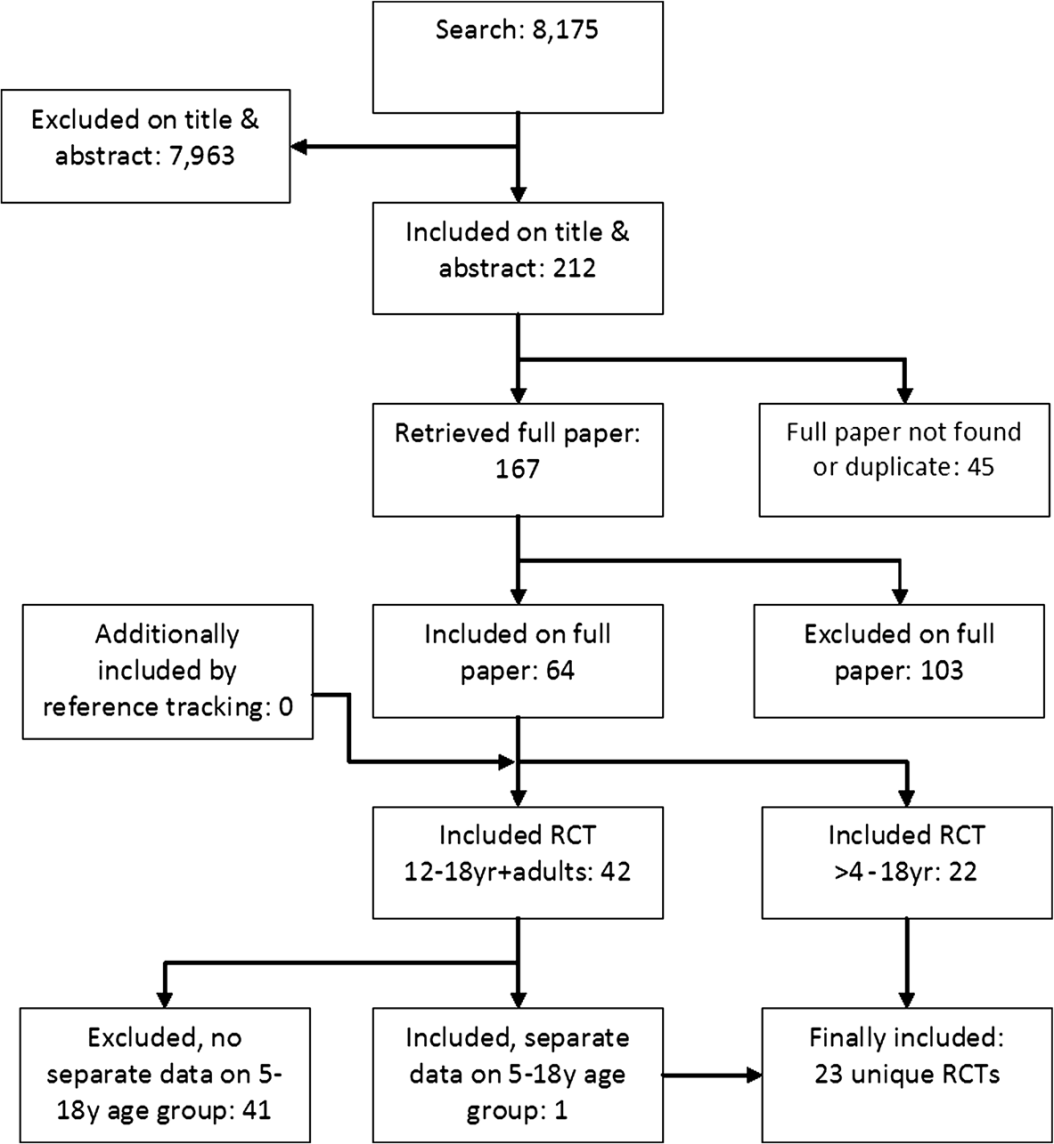
**Flowchart from database searches to inclusion of the trials.** 7,152 of the 8,175 references were excluded because they did not fulfil the inclusion criteria. Main reasons for exclusion were: reference was not a trial, wrong age group or no separate data for < 18 year olds, wrong dosage, not asthma, follow up duration < 4 weeks or cross-over design.

**Figure 2:**
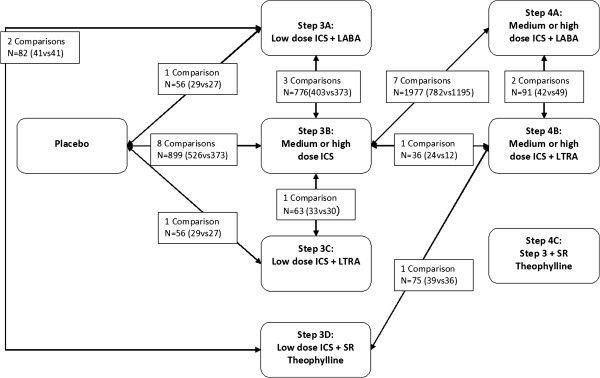
**The network of included trials in GINA step 3&4.** ICS = Inhaled Corticosteroids; LABA = Long–acting β2-adrenoceptor agonists; SR= Sustained release. The arrows represent the direct comparisons found in the included RCTs, including the number of RCTs and total number of participants. Except for SR theophylline as add-on to step 3, all treatments are directly or indirectly connected to each other. An example of an indirect comparison replacing a non-existent comparison is step 3A to 4A, through 3A to 3B, and 3B to 4A.

The comprehensive literature search yielded 8,175 references (see Figure 1). We retrieved 200 as full text articles, representing 160 unique studies. Reference tracking of the GINA guideline, systematic reviews and included references did not yield additional references. Twenty-three trials, conducted between 1984 and 2010, met the inclusion criteria and were included [6-10,19-36]. Additional file 4 shows the study characteristics of the 23 trials with 4,129 patients ranging in age from 4 to 18 years; we included 6 trials with a lower age range of 4 years, but with a mean or median between 5 and 18 years [6,9,29,31,32,36]. Figure 2 shows the network of direct and indirect comparisons. There are 28 theoretically possible pair-wise comparisons: all 7 GINA options versus placebo and 21 head-to-head comparisons, 3A versus 3B, …, 3A versus 4C, and, taking the other options as a starting point, all the way to 4B versus 4C. The arrows represent the ten actually published direct comparisons. The white boxes show the number of RCTs and total number of participants for each comparison. In total, we found seven different head-to-head comparisons (with between 1 and 7 studies per comparison) and 3 different comparisons with placebo (with between 1 and 8 studies). All indirect comparisons were possible, except for comparisons with GINA 4c (medium dose ICS+theophylline as add-on to step 3), which is not connected to the network. An example of a possible indirect comparison replacing a non-existent direct comparison is step 3A versus 4A via 3B. A more complicated example is 3D versus 4A via 4B and 3B. An example where both direct and indirect comparisons exist would be 3B vs 3C, namely, direct via a N=63 trial, and indirect via N=955(899(3B vs Placebo)+56(Placebo vs 3C)) participants. The latter example illustrates how NMA may add strength to scarcely investigated direct comparisons. Figure 2 shows that 3A versus 3B (N=776), 3B versus placebo (N=899) and 3B versus 4A (N=1977) are relatively well researched, while most other comparisons depend on weak statistical evidence. However, there are some comparisons that benefit from the relatively strong 3B versus placebo connection, for example, 3A versus 3B, and 3B versus 3C.

The high number of question marks (138/207, or 67%) in Table 2 indicates that incomplete, unclear or non-reporting hampered thorough quality assessment. Eleven out of 23 trials reported on compliance, while only 4 reported on blinding of the physician or how missing values were dealt with.

### Outcome measurements

We found enormous variation in choice of outcome measures and how they were reported. Twenty-one studies reported FEV_1_, but variation in methods of reporting was quite extreme (Table 3). None of the studies reported IPD. One study reported the method of converting “liters” to “percentage of predicted” (e.g. Quanjer, Zapletal, Polgar or Hankinson) [32]. Thus, although FEV_1_ is an outcome that 21/23 studies reported in some form, the results could not be compared straightforwardly, nor pooled. Pooling outcomes on asthma symptoms, the second best, was also not possible (see Additional file 5).

**Table 3:** **Reporting method of FEV**_**1 **_**for all included trials for each scale (liter or % of predicted) and statistical method of reporting, at baseline (T**_**0**_**) and endpoint (T**_**e**_**)**

**Scale of FEV**_**1**_	**Outcome summary measure**	**Reference number of study reporting summary measure at T**_**0**_^a^	**Reference number of study reporting summary measure at T**_**e**_^**b**^
**Liter**	Mean		13, 15, 19
	Mean change		10, 14, 18
	Mean + SE or SD	8, 10, 11, 16, 18, 22, 23	8, 16
	Mean change + SE or SD		23
	Mean + range	14, 15, 16, 19	16
	P-value versus another arm		10, 14, 15, 18, 19, 23
	Difference between arms + 95%CI		13, 23
**% of predicted**	Mean		13
	Mean change		11, 12, 18
	Mean + SE or SD	2, 3, 5, 6, 7, 9, 10, 11, 16, 17, 18, 20, 21, 22	2, 3, 6, 7, 9, 16, 17, 20
	Mean change + SE or SD		
	Mean + range	12, 13, 14, 15, 16, 17, 19	16
	P-value versus another arm		11, 12, 18
	Difference between arms + 95%CI		13, 17

^a^T_0_ = baseline of the trial.

^b^T_e_ = endpoint of the trial, varying from 4 to 56 weeks.

Reporting method of all included trials for each scale (litre or % of predicted) and statistical method of reporting, at baseline (T_0_) and endpoint (T_e_).

### Attempts to salvage the situation

FEV_1−_values depend on sex, age, and height. FEV_1_-values are usually not normally distributed and extreme values occur, skewing the mean [31]. Besides differences in reporting of litres and percentages of predicted, the mix of outcome measures and statistical details was often reported unsystematically and awkwardly. Intra-arm differences instead of between-arm differences were often reported, while descriptive statistics (standard deviation, range) were used where inferential statistics (standard errors, confidence intervals) were needed. These inconsistencies or mistakes thwarted our attempts at pooling of results and made a sensible summary difficult altogether. We contacted authors or sponsors for more details (e.g. summaries of patient characteristics for height, IPD, alternative outcome measurements such as the mean difference between the groups with corresponding standard errors, different time point of follow-up) to allow expressing the results on identical scales. Unfortunately, only in four instances we received additional information through these personal communications [7,8,22,34].

We used those trials that reported both FEV_1_(l) and FEV_1_%_pred_ to directly compare our simulation results with those empirically measured in these trials, and found that, regrettably, they were very different. In particular, the ranges of the results were much narrower than the empirically measured percentages of predicted. In some cases, results from our conversion method were opposite to the true results (simulated result of FEV_1_>100% of predicted versus an observed result of FEV_1_<100% of predicted) [6,30,31]. Because of these considerable and irresolvable discrepancies, we decided that formal meta-analysis seemed irresponsible. This decision was made easier by the deficient reporting and potentially low methodological quality of many trials.

### Descriptive findings of key trials

Since we are not able to pool the data and establish an evidence based ranking of effectiveness of drug treatments in GINA steps 3 and 4, we describe the main findings from the trials of the most frequently compared interventions (>2 trials/comparison), including over 100 patients per intervention group and having a follow up of at least 8 weeks. Many different outcomes are reported. However, in the trial descriptions below we restrict our focus to the following clinically most relevant ones: number of exacerbations, level of control, reliever medication use, symptom score, frequency of night-time awakening, quality of life, FEV_1,_ hyperresponsiveness (PC_20_-FEV_1_) and PEF. The only interventions compared more than two times were steps 3A versus 3B, that is, adding a LABA to a low dose of ICS or increasing the dose of ICS, and 3B versus 4A, that is, adding a LABA to medium or high dose ICS.

#### Step 3A versus 3B

Three trials [6,32,36] published between 2006 and 2009 compared a medium or high dose ICS to low dose ICS plus LABA: Gappa et al. (age 4-16 years; n=138 and 145; QA-score=4/9), Bisgaard et al. (age 4-11 years; N=117, 118 and 106; QA-score=2/9) and De Blic et al. (age 4-11 years; N=150 and 153; QA-score=7/9). Bisgaard et al. in a 3-armed trial, compared a fixed low dose of ICS plus LABA, a non-fixed low dose (‘SMART’) of ICS plus a LABA and a medium dose of ICS. The authors claim significant effects from the SMART ‘regimen compared to medium ICS or fixed dose. But according to the GINA classification the two ICS plus LABA regimens are ‘GINA 3A’ and for the purpose of this review we see no additional value of comparing between these two GINA 3A arms. We excluded the results of the non-fixed-dose group from this discussion. Because participants in the non-fixed dose group were allowed to take additional study medication (ICS+LABA), only a mean number as-needed-use inhalations (daytime: 0.49 & nighttime: 0.09) in this group is reported. Regrettably, no range or standard deviation is mentioned. Therefore it is possible that some participants were in fact treated according to GINA 4A.

As presented in Table 4, Gappa et al. as well as Bisgaard et al. found that adding LABA (3A) improved the level of control statistically significantly more than doubling the dose of ICS (3B). However, the trial by De Blic et al. was unable to confirm this. Gappa et al. and De Blic et al. found a statistically significantly lower use of rescue medication in the LABA group after 12 weeks compared to the ICS group, but Bisgaard et al. found no difference. Only Bisgaard et al. found a significantly better improvement of the symptom score in the LABA group compared to the ICS group.

**Table 4:** Reported significant differences between intervention groups per trial

**Study**	**3A-3B**			**3B-4A**		
**Outcome**	**Gappa**[**32**]**Differences between groups (95%CI)**	**Bisgaard**[**6**]**Differences between groups (p)**	**De Blic**[**36**]**Differences between groups (95%CI; p)**	**Tal**[**29**]**Differences between groups (95%CI)**	**Morice**[**30**]**Differences between groups (95%CI; p)**	**Pohunek**[**31**]**Differences between groups (p)**
Number of exacerbations	n.a.	n.d.	n.d.	n.a.	n.a.	n.a.
Level of control	p=0.02^a^	9.8(0.047)^b^	n.d.	n.d.	n.d.	n.d.
Reliever medication use/reliever free days^c^	8.7(1.2-16.3)	n.d.	1.4(0.0-3.4; 0.025)	n.d.	n.d.	n.d.
Symptom score (day&night)	n.d.	0.27(0.024)	n.a.	n.d.	n.d.	n.d.
nighttime awakening	n.a.	n.d.	n.d.	n.d.	n.d.	n.d.
Quality of life	n.a.	n.a.	n.a.	n.a.	n.d.	n.d.
Lung function (FEV_1_)	n.d.	n.d.	n.d.	3.75(1.1-6.4)^d^	n.a.	0.08(<0.01)^e^ & 0.06(<0.001)^e^
Hyperresponsiveness (PC_20_-FEV_1_)	n.a.	n.a.	n.a.	n.a.	n.a.	n.a.
morning PEF	6.1(1.8-10.4)^d^	n.d.	7.6(1.7-13.5)^e^	3.77(1.84-5.7)^d^	9.5(4.2-14.9; <0.001)^e^ & 10.3(5.0-15.6; <0.001)^e,f^	15(<0.001)^e^ & 6(<0.001)^e,f^

^a^ nr of weeks with successful asthma control.

^b^ asthma-control days (%).

^c^ Percentage of days without rescue medication.

^d^ % of predicted.

^e^ liter.

^f^ For some studies 2 results are presented because three groups were compared in the trial.

n.a. = not available.

n.d. = no statistically significant differences.

Overall, these larger trials seem to support the view that there is a larger benefit from adding LABA to a low dose of ICS than from doubling the dose of ICS (See Table 4).

#### Step 3b versus 4A

Seven trials [8,10,21,29-31,35] published between 1995 and 2007 compared a medium or high dose of ICS to a medium or high dose of ICS plus LABA. Three trials [29-31] contained more than 100 patients per group and had a follow up of more than 8 weeks: Tal et al. (age 4-17 years; N=138 and 148; QA-score=3/9), Pohunek et al. (age 4-11 years; N=213, 201 and 216; QA-score=1/9) and Morice et al. (age 6-11 years; N=212, 203 and 207; QA-score=3/9). None of the trials found statistically significant differences between the groups on number of exacerbations, level of control, use of rescue medication, symptoms scores, nighttime awakenings or quality of life. As presented in Table 4, Tal et al. and Pohunek et al. both found a statistically significantly larger benefit on FEV_1_ in the LABA group after 12 weeks compared to the ICS group. All three trials found statistically significant differences in favour of LABA on morning PEF after 12 weeks.

The three studies described here seem to support the idea that adding LABA to medium dose ICS is slightly more effective, although as measured by lung function only.

## Discussion

We tried to synthesize the evidence for GINA step 3&4 recommendations for 5 to 18 year-olds with moderate persistent asthma. Our aim was to rank the 21 different GINA treatment options as to their effectiveness using standard systematic review methods extended by network meta-analytic techniques.

In principle, the situation looked favourable for network meta-analysis, with RCTs on six out of seven interventions either against placebo or head-to-head (Figure 2). Lack of direct comparisons, for example GINA 3C versus 4A, could have been compensated by indirect comparisons, for example through GINA 3B and placebo. Only theophylline was disconnected to the network of trials as we found no trials in this age group.

Due to extremely different choices trialists made on outcome reporting methods, we had to abandon attempts at meta-analysis. Apart from embarking on a set of concerted new trials in this area, which may take years to complete, a potentially quicker way to salvage the situation with existing data may be joint action among sponsors and trialists of existing trials to aggregate their raw data to inform an IPD meta-analysis [42,43]. The authors of this review would be more than happy to support such an endeavour, thereby achieving this review’s original aim. Such an exercise would depend also on the results of additional trialist-provided information on trial quality, since pooling of very low quality data is unattractive. This brings us to the next point. We assessed the risk of bias in the included trials on a 9-item methodological quality checklist. We scored “?” if the risk of bias seemed hard to determine. We scored 138/207 “?”, and this is largely due to partial, unclear or non-reporting (see Table 2). Adoption and enforcement of the CONSORT statement should become a priority for trialists and journals alike [44].

After criticizing some of the outcome reporting methods, let us consider the strengths and limitations of our own work. We comprehensively searched the literature and tried to minimize the risk of missing RCTs by tracking the references of the GINA-guideline, included RCTs and relevant systematic reviews [45-47]. However, these efforts yielded no additional relevant references. We performed all major steps, except the extraction of the quantitative data in duplicate. Furthermore, our team had expertise on all aspects of a systematic review: clinical librarian, biostatistician, physician-epidemiologist, two general practitioners, a trainee general practitioner, and a paediatric pulmonologist. Nevertheless, our review is no exception in that it may have been affected by suppression of negative trial results, or publication bias [48].

As far as we are aware, a network meta-analysis on this subject would have been novel. The majority of the meta-analyses performed on these treatment options are combined for paediatric and adult patients. In 2003, Bisgaard analyzed the effect of long–acting β_2_-adrenoceptor agonists (LABA) on the asthma exacerbation rate in paediatric patients in a review of eight randomized trials [46]. All trials compared a LABA with a SABA or placebo in children on inhaled corticosteroids and reported on exacerbations or asthma-related hospitalizations in asthmatic children. Bisgaard, while providing the spectrum of relative risks, refrained from formal meta-analysis, because of differences in patient populations, comparators, study design and duration, and definitions of asthma exacerbation. He concluded that there is no evidence in the existing paediatric literature that LABA protects against asthma exacerbations, even when used as an add-on therapy to ICS.

In line with our view that firm evidence to guide step-up therapy is lacking, Lemanske et al. performed the BADGER trial, a three-period-cross-over trial in children eligible for GINA step 3 [49]. The BADGER trial is clearly relevant to the topic of this review. The study addresses the research question which of the three medication options (doubling the dose of the inhaled corticosteroid, adding LABA or LTRA) should be the first choice of treatment in step 3 of the guidelines. Because of its importance to the research question of this review, we will discuss it in some more detail. The BADGER investigators assigned 182 children, from 6 to 17 years of age with uncontrolled asthma, despite receiving a low dose ICS to receive each of three blinded step-up therapies, corresponding with GINA step 3A, 3B and 3C, in random order for a period of 16 weeks each. Several clinical and physical aspects were measured, including the need for oral prednisone, an asthma control test and FEV_1_. Main outcome was that overall, LABA as add-on (GINA 3A) performed better than increasing ICS dose (GINA 3B) or adding LTRA (GINA 3C). Furthermore, subgroup analyses were performed to predict the direction of the patterns of differential response, primary on baseline values of PC_20_, Asthma Control Test scores and genotype, and, post hoc, on demographic and physiological characteristics. The only significant (p=0.009) predictor was the baseline Asthma Control Test scores (</≥19) on the probability of the best response to LABA step-up.

Strengths of the BADGER trial are the topical research questions and relevant outcomes measures. Furthermore, sensitivity analyses were performed to assess bias, for example seasonal differences. However, the treatment period-specific results were not reported separately, which was the main reason why we could not use the trial in this review with network meta-analysis. In addition, the study is hampered by the cross-over design with possible carry-over effects of ICS treatment. A wash-out period of four weeks makes using the second and third treatment periods hazardous due to unquantifiable carry-over effects [16]. Carry-over effect of ICS would have improved the treatment effects of adding LABA or LTRA. Furthermore, post hoc analysis with relatively small subgroups already raised much discussion and suggests hypotheses that need more research in studies with a different design [50-54].

Although GINA provides us with treatment recommendations, steps 3&4 are still not based on sound evidence. For patients, their parents, and physicians alike, uncertainty about the best treatment remains. New trials should focus on add-on therapy to ICS in children. Ongoing and new RCTs will be part of meta-analysis in a few years. To interpret individual studies, consensus about design and reporting of outcome measurements for RCTs would provide a much better evidence base for the future. In 2009 an official American Thoracic Society/European Respiratory Society statement, about standardizing endpoints for clinical asthma trials and clinical practice was published [55]. A taskforce formulated recommendations of assessment for the design, conduct and evaluation of asthma trials for clinicians, researchers, and other relevant groups. These recommendations form an excellent starting point for harmonization of outcome measures and accompanying inferential statistical measures in RCTs and other comparative effectiveness research. As far back as 1992, Tugwell and Boers introduced a solution for Rheumatoid Arthritis Clinical Trials, OMERACT (“Outcome Measures in Rheumatoid Arthritis Clinical Trials”) [56]. OMERACT, an international informal network, strives to improve outcome measurement through a data driven, iterative consensus process involving relevant stakeholder groups. This type of initiative would be welcome in asthma research too.

Another solution may be prospective meta-analysis (PMA) [17,42]. PMA meta-analyses RCTs, preferably by using IPD, that were identified, evaluated and determined to be eligible for the meta-analysis before the results of any of those studies become known. PMA was developed to overcome some of the problems of normal (retrospective) meta-analyses, mainly to enable hypotheses to be specified a priori and ignorant of the results of individual trials. Ideally, PMA provides standardization of clinical trial procedures, such as study design and data collection methods, by using, for example, the same instruments and the same time points for measuring outcomes.

## Conclusion

Due to extreme variation in choice of outcome measures and their reporting, firm evidence-based ranking of effectiveness of the treatment options in GINA 3&4 for 5 to 18 year-olds based on evidence from randomized trials is currently impossible. Implementation of the recommendations issued by the recent ATS/ERS taskforce on measures of asthma control in RCTs is urgent.

## Competing interests

The authors declare that they have no competing interests.

## Authors’ contribution

LvdM had the lead, participated in the design of the study, carried out the protocol, literature search, reviewed the articles, extracted data and drafted the manuscript. EL assisted with the literature search, was second reviewer, extracted data and helped revise the manuscript critically. RG participated in the design of the study, performed the statistical analysis, drafted the part of the statistical analysis of the manuscript and revised the manuscript critically. JM participated in preparing the data for statistical analysis and revised the manuscript critically. PB participated in the design of the study and revised the manuscript critically. WvA conceived the study, participated in the design of the study and revised the manuscript critically. GtR conceived the study, participated in the design of the study and coordination, participated in the statistical analysis and helped to draft the manuscript. All authors read and approved the manuscript.

## Glossary of some terms

Level A evidence: a substantial number of well designed RCTs exist, with substantial numbers of participants, in the recommended population, with consistent patterns of findings (1).

Level B evidence: few RCTs exist; they are small in size, undertaken in a different population or results are not consistent (1).

Carry-over effect: the persistence of a treatment applied in one period in a subsequent period of treatment (2).

## Pre-publication history

The pre-publication history for this paper can be accessed here:

http://www.biomedcentral.com/1471-2466/12/63/prepub

## Supplementary Material

Additional file 1 Search Strategy. Click here for file

Additional file 2 **Appendix 2.** Medication options GINA step 3&4 for 5-18 year olds. Click here for file

Additional file 3 **Appendix 3.** Statistical Analysis – The modeling used for FEV_1_%_pred._ Click here for file

Additional file 4 **Appendix 4.** Study characteristics. Click here for file

Additional file 5 **Appendix 5.** Measured and reported Asthma Symptoms and Inhaled β2-agonist use per trial. Click here for file
