# Supplementary material for: A systematic review with attempted network meta-analysis of asthma therapy recommended for five to eighteen year olds in GINA steps three and four
Source: BMC Pulm Med. 2012 Oct 15;12:63. doi: 10.1186/1471-2466-12-63 (PMC3582530; doi:10.1186/1471-2466-12-63)
Supplement: Additional file 1 — Search Strategy. [file 1471-2466-12-63-S1.doc]

**Search Strategy**

**PubMed**

<http://www.ncbi.nlm.nih.gov/pubmed/>. Date last accessed. February 4 2010.

(((("Infant"[Mesh] OR infant*[tiab] OR "Child"[Mesh] OR child*[tiab] OR "Child, Preschool"[Mesh] OR Preschool Child*[tiab] OR "Adolescent"[Mesh] OR adolescen*[tiab])) AND (("Asthma"[Mesh] OR asthma[tiab]))) AND (("Anti-Asthmatic Agents"[Mesh] OR "Anti-Asthmatic Agents "[Pharmacological Action] OR "Administration, Inhalation"[Mesh] OR "Nebulizers and Vaporizers"[Mesh] OR "montelukast "[Substance Name] OR montelukast[tiab] OR "Leukotriene Antagonists"[Mesh] OR "Leukotriene Antagonists "[Pharmacological Action] OR "Leukotrienes"[Mesh] OR leucotriene[tiab] OR "pranlukast "[Substance Name] OR "Theophylline"[Mesh] OR theophylline[tiab] OR theophyllamine[tiab] OR "Aminophylline"[Mesh] OR aminophylline[tiab] OR formoterol[tiab] OR salmeterol[tiab] OR "zafirlukast "[Substance Name] OR "zileuton "[Substance Name] OR inhaled corticosteroid*[tiab] OR "Budesonide"[Mesh] OR "fluticasone "[Substance Name] OR "fluticasone, salmeterol drug combination"[Substance Name] OR "ciclesonide "[Substance Name] OR "flunisolide "[Substance Name] OR "mometasone furoate "[Substance Name] OR "Triamcinolone Acetonide"[Mesh] OR dry powder inhaler*[tiab] OR "Metered Dose Inhalers"[Mesh] OR Sustained-release tablet*[tiab] OR ICS[tiab] OR "Beclomethasone"[Mesh] OR "Adrenergic beta-Agonists"[Substance Name] OR Long-acting beta-2-agonist*[tiab] OR "Adrenergic beta-Agonists"[Mesh] OR "Sympathomimetics"[Mesh] OR "Sympathomimetics "[Pharmacological Action] OR LABA[tiab] OR "formoterol"[Substance Name] OR "salmeterol"[Substance Name] OR Sustained-release tablet*[tiab] OR "albuterol"[TIAB] OR "albuterol"[MeSH Terms] OR Salbutamol[tiab] OR "tablets"[MeSH Terms] OR tablet*[tiab] OR "Terbutaline"[Mesh] OR terbutaline[tiab] OR (("budesonide"[MeSH Terms] OR Budesonide[tiab]) AND ("formoterol"[Substance Name] OR formoterol[tiab])) OR (("fluticasone"[Substance Name] OR Fluticasone[tiab]) AND ("salmeterol"[Substance Name] OR salmeterol[tiab]))))) AND (((("Clinical Trial "[Publication Type] OR "Clinical Trials as Topic"[Mesh] OR "Randomized Controlled Trial "[Publication Type] OR "Randomized Controlled Trials as Topic"[Mesh] OR "Random Allocation"[Mesh] OR "Single-Blind Method"[Mesh] OR "Double-Blind Method"[Mesh] OR "Cross-Over Studies"[Mesh] OR "Placebos"[Mesh] OR RCT[tiab] OR Random allocation[tiab] OR Randomly allocated[tiab] OR Single blind*[tiab] OR Double blind*[tiab] OR triple blind*[tiab] OR placebo*[tiab] OR "Prospective Studies"[Mesh])) NOT (("Case Reports "[Publication Type] OR "Letter "[Publication Type]))))

###

### EMBASE.

<http://ovidsp.tx.ovid.com/sp-2.3/ovidweb.cgi?>. Date last accessed: February 4 2010.

| 1 | child/ or child*.ti,ab. or preschool child/ or preschool child*.ti,ab. or infant*.ti,ab. or infant/ or Adolescent/ or adolescen*.ti,ab. |
| --- | --- |
| 2 | exp Asthma/ or asthma.ti,ab. |
| 3 | exp Antiasthmatic Agent/ or inhalational drug administration/ or exp nebulizer/ or Montelukast/ or montelukast.ti,ab. or exp Leukotriene Receptor Blocking Agent/ or exp Leukotriene/ or leucotriene.ti,ab. or Pranlukast/ or Theophylline/ or theophylline.ti,ab. or theophyllamine.ti,ab. or Aminophylline/ or Zafirlukast/ or Zileuton/ or inhaled corticosteroid*.ti,ab. or exp BUDESONIDE PLUS FORMOTEROL/ or exp BUDESONIDE/ or exp FLUTICASONE PROPIONATE PLUS SALMETEROL/ or exp FLUTICASONE/ or Ciclesonide/ or Flunisolide/ or Mometasone Furoate/ or exp Triamcinolone Acetonide/ or dry powder inhaler*.ti,ab. or inhaler/ or metered dose inhaler/ or powder inhaler/ or exp Sustained Release Preparation/ or exp Beclometasone/ or exp Beta Adrenergic Receptor Stimulating Agent/ or Beta 2 Adrenergic Receptor Stimulating Agent/ or long-acting beta-2 agonist*.ti,ab. or exp Adrenergic Receptor Stimulating Agent/ or sympathomimetic*.ti,ab. or LABA.ti,ab. or ICS.ti,ab. or exp FORMOTEROL/ or formoterol.ti,ab. or exp SALMETEROL/ or salmeterol.ti,ab. or exp FLUTICASONE PROPIONATE PLUS SALMETEROL/ or sustained release tablet*.ti,ab. or exp Salbutamol/ or albuterol.ti,ab. or salbutamol.ti,ab. or exp TABLET/ or tablet*.ti,ab. or exp TERBUTALINE/ or terbutaline.ti,ab. |
| 4 | (Clinical trial/ or Randomized controlled trial/ or Randomization/ or Single blind procedure/ or Double blind procedure/ or Crossover procedure/ or Placebo/ or Randomi?ed controlled trial$.tw. or Rct.tw. or Random allocation.tw. or Randomly allocated.tw. or Allocated randomly.tw. or (allocated adj2 random).tw. or Single blind$.tw. or Double blind$.tw. or ((treble or triple) adj blind$).tw. or Placebo$.tw. or Prospective study/) not (Case study/ or Case report.tw. or Abstract report/ or letter/) |
| 5 | 1 and 2 and 3 and 4 |

###

### CINAHL.

<http://web.ebscohost.com/ehost/search?vid=1&hid=7&sid=1b408965-8b73-4a1c-9b6b-0262a4a503e4@sessionmgr12>. Date last accessed: February 4 2010-02-25

| # | Query | Limiters/Expanders | Last Run Via |
| --- | --- | --- | --- |
| S54 | S34 and S51 | Limiters - Published Date from: 20090601-20100231  Expanders - Apply related words; Also search within the full text of the articles  Search modes - Boolean/Phrase | Interface - EBSCOhost  Search Screen - Advanced Search  Database - CINAHL Plus with Full Text |
| S53 | S34 and S51 | Expanders - Apply related words; Also search within the full text of the articles  Search modes - Boolean/Phrase | Interface - EBSCOhost  Search Screen - Advanced Search  Database - CINAHL Plus with Full Text |
| S52 | S34 and S51 | Expanders - Apply related words; Also search within the full text of the articles  Search modes - Boolean/Phrase | Interface - EBSCOhost  Search Screen - Advanced Search  Database - CINAHL Plus with Full Text |
| S51 | S49 not S50 | Expanders - Apply related words; Also search within the full text of the articles  Search modes - Boolean/Phrase | Interface - EBSCOhost  Search Screen - Advanced Search  Database - CINAHL Plus with Full Text |
| S50 | S48 or S47 | Expanders - Apply related words; Also search within the full text of the articles  Search modes - Boolean/Phrase | Interface - EBSCOhost  Search Screen - Advanced Search  Database - CINAHL Plus with Full Text |
| S49 | S46 or S45 or S44 or S43 or S42 or S41 or S40 or S39 or S38 or S37 or S36 or S35 | Expanders - Apply related words; Also search within the full text of the articles  Search modes - Boolean/Phrase | Interface - EBSCOhost  Search Screen - Advanced Search  Database - CINAHL Plus with Full Text |
| S48 | (MH "Writing") | Expanders - Apply related words; Also search within the full text of the articles  Search modes - Boolean/Phrase | Interface - EBSCOhost  Search Screen - Advanced Search  Database - CINAHL Plus with Full Text |
| S47 | ( (case study or case report or abstract report) or (MH "Case Studies") ) or letter | Expanders - Apply related words; Also search within the full text of the articles  Search modes - Boolean/Phrase | Interface - EBSCOhost  Search Screen - Advanced Search  Database - CINAHL Plus with Full Text |
| S46 | (MH "Prospective Studies") | Expanders - Apply related words; Also search within the full text of the articles  Search modes - Boolean/Phrase | Interface - EBSCOhost  Search Screen - Advanced Search  Database - CINAHL Plus with Full Text |
| S45 | triple blind* or treble blind* | Expanders - Apply related words; Also search within the full text of the articles  Search modes - Boolean/Phrase | Interface - EBSCOhost  Search Screen - Advanced Search  Database - CINAHL Plus with Full Text |
| S44 | allocated randomly or Single blind* or double blind* | Expanders - Apply related words; Also search within the full text of the articles  Search modes - Boolean/Phrase | Interface - EBSCOhost  Search Screen - Advanced Search  Database - CINAHL Plus with Full Text |
| S43 | RCT or random allocation or randomly allocated | Expanders - Apply related words; Also search within the full text of the articles  Search modes - Boolean/Phrase | Interface - EBSCOhost  Search Screen - Advanced Search  Database - CINAHL Plus with Full Text |
| S42 | placebo* | Expanders - Apply related words; Also search within the full text of the articles  Search modes - Boolean/Phrase | Interface - EBSCOhost  Search Screen - Advanced Search  Database - CINAHL Plus with Full Text |
| S41 | (MH "Placebos") | Expanders - Apply related words; Also search within the full text of the articles  Search modes - Boolean/Phrase | Interface - EBSCOhost  Search Screen - Advanced Search  Database - CINAHL Plus with Full Text |
| S40 | (MH "Crossover Design") | Expanders - Apply related words; Also search within the full text of the articles  Search modes - Boolean/Phrase | Interface - EBSCOhost  Search Screen - Advanced Search  Database - CINAHL Plus with Full Text |
| S39 | (MH "Single-Blind Studies") or (MH "Double-Blind Studies") or (MH "Triple-Blind Studies") | Expanders - Apply related words; Also search within the full text of the articles  Search modes - Boolean/Phrase | Interface - EBSCOhost  Search Screen - Advanced Search  Database - CINAHL Plus with Full Text |
| S38 | (MH "Single-Blind Studies") | Expanders - Apply related words; Also search within the full text of the articles  Search modes - Boolean/Phrase | Interface - EBSCOhost  Search Screen - Advanced Search  Database - CINAHL Plus with Full Text |
| S37 | Randomization or random allocation* | Expanders - Apply related words; Also search within the full text of the articles  Search modes - Boolean/Phrase | Interface - EBSCOhost  Search Screen - Advanced Search  Database - CINAHL Plus with Full Text |
| S36 | randomized controlled trial* or randomised controlled trial* | Expanders - Apply related words; Also search within the full text of the articles  Search modes - Boolean/Phrase | Interface - EBSCOhost  Search Screen - Advanced Search  Database - CINAHL Plus with Full Text |
| S35 | (MH "Clinical Trials") | Expanders - Apply related words; Also search within the full text of the articles  Search modes - Boolean/Phrase | Interface - EBSCOhost  Search Screen - Advanced Search  Database - CINAHL Plus with Full Text |
| S34 | S33 and S7 and S6 | Expanders - Apply related words; Also search within the full text of the articles  Search modes - Boolean/Phrase | Interface - EBSCOhost  Search Screen - Advanced Search  Database - CINAHL Plus with Full Text |
| S33 | S32 or S31 | Expanders - Apply related words; Also search within the full text of the articles  Search modes - Boolean/Phrase | Interface - EBSCOhost  Search Screen - Advanced Search  Database - CINAHL Plus with Full Text |
| S32 | ( (fluticasone) or (MH "Fluticasone") ) and ( (salmeterol) or (MH "Salmeterol") ) | Expanders - Apply related words; Also search within the full text of the articles  Search modes - Boolean/Phrase | Interface - EBSCOhost  Search Screen - Advanced Search  Database - CINAHL Plus with Full Text |
| S31 | S30 or S29 or S28 or S27 or S26 or S25 or S24 or S23 or S22 or S21 or S20 or S19 or S18 or S17 or S16 or S15 or S14 or S13 or S12 or S11 or S10 or S9 or S8 | Expanders - Apply related words; Also search within the full text of the articles  Search modes - Boolean/Phrase | Interface - EBSCOhost  Search Screen - Advanced Search  Database - CINAHL Plus with Full Text |
| S30 | (Terbutaline) or (MH "Terbutaline") | Expanders - Apply related words; Also search within the full text of the articles  Search modes - Boolean/Phrase | Interface - EBSCOhost  Search Screen - Advanced Search  Database - CINAHL Plus with Full Text |
| S29 | ( (albuterol) or (MH "Albuterol") ) | Expanders - Apply related words; Also search within the full text of the articles  Search modes - Boolean/Phrase | Interface - EBSCOhost  Search Screen - Advanced Search  Database - CINAHL Plus with Full Text |
| S28 | (tablet*) or (MH "Tablets") | Expanders - Apply related words; Also search within the full text of the articles  Search modes - Boolean/Phrase | Interface - EBSCOhost  Search Screen - Advanced Search  Database - CINAHL Plus with Full Text |
| S27 | formoterol or salmeterol or salbutamol | Expanders - Apply related words; Also search within the full text of the articles  Search modes - Boolean/Phrase | Interface - EBSCOhost  Search Screen - Advanced Search  Database - CINAHL Plus with Full Text |
| S26 | (MH "Sympathomimetics") or LABA or ICS | Expanders - Apply related words; Also search within the full text of the articles  Search modes - Boolean/Phrase | Interface - EBSCOhost  Search Screen - Advanced Search  Database - CINAHL Plus with Full Text |
| S25 | (MH "Beclomethasone") or (MH "Adrenergic Beta-Agonists") or Long-acting beta-2-agonist* | Expanders - Apply related words; Also search within the full text of the articles  Search modes - Boolean/Phrase | Interface - EBSCOhost  Search Screen - Advanced Search  Database - CINAHL Plus with Full Text |
| S24 | dry powder inhaler* or Metered Dose Inhaler* or ( (Sustained-release tablet*) or (MH "Delayed-Action Preparations") ) | Expanders - Apply related words; Also search within the full text of the articles  Search modes - Boolean/Phrase | Interface - EBSCOhost  Search Screen - Advanced Search  Database - CINAHL Plus with Full Text |
| S23 | (Triamcinolone Acetonide) or (MH "Triamcinolone") | Expanders - Apply related words; Also search within the full text of the articles  Search modes - Boolean/Phrase | Interface - EBSCOhost  Search Screen - Advanced Search  Database - CINAHL Plus with Full Text |
| S22 | mometasone furoate | Expanders - Apply related words; Also search within the full text of the articles  Search modes - Boolean/Phrase | Interface - EBSCOhost  Search Screen - Advanced Search  Database - CINAHL Plus with Full Text |
| S21 | flunisolide | Expanders - Apply related words; Also search within the full text of the articles  Search modes - Boolean/Phrase | Interface - EBSCOhost  Search Screen - Advanced Search  Database - CINAHL Plus with Full Text |
| S20 | ciclesonide | Expanders - Apply related words; Also search within the full text of the articles  Search modes - Boolean/Phrase | Interface - EBSCOhost  Search Screen - Advanced Search  Database - CINAHL Plus with Full Text |
| S19 | (FLUTICASONE and SALMETEROL) or (MH "Salmeterol") or (MH "Fluticasone") | Expanders - Apply related words; Also search within the full text of the articles  Search modes - Boolean/Phrase | Interface - EBSCOhost  Search Screen - Advanced Search  Database - CINAHL Plus with Full Text |
| S18 | budesonide and formoterol | Expanders - Apply related words; Also search within the full text of the articles  Search modes - Boolean/Phrase | Interface - EBSCOhost  Search Screen - Advanced Search  Database - CINAHL Plus with Full Text |
| S17 | (MH "Budesonide") or (MH "Fluticasone") | Expanders - Apply related words; Also search within the full text of the articles  Search modes - Boolean/Phrase | Interface - EBSCOhost  Search Screen - Advanced Search  Database - CINAHL Plus with Full Text |
| S16 | zileuton or Inhaled Corticosteroid* | Expanders - Apply related words; Also search within the full text of the articles  Search modes - Boolean/Phrase | Interface - EBSCOhost  Search Screen - Advanced Search  Database - CINAHL Plus with Full Text |
| S15 | zafirlukast | Expanders - Apply related words; Also search within the full text of the articles  Search modes - Boolean/Phrase | Interface - EBSCOhost  Search Screen - Advanced Search  Database - CINAHL Plus with Full Text |
| S14 | ( (Theophylline) or (MH "Theophylline") ) or theophyllamine or Aminophylline | Expanders - Apply related words; Also search within the full text of the articles  Search modes - Boolean/Phrase | Interface - EBSCOhost  Search Screen - Advanced Search  Database - CINAHL Plus with Full Text |
| S13 | MH montelukast or montelukast | Expanders - Apply related words; Also search within the full text of the articles  Search modes - Boolean/Phrase | Interface - EBSCOhost  Search Screen - Advanced Search  Database - CINAHL Plus with Full Text |
| S12 | pranlukast | Expanders - Apply related words; Also search within the full text of the articles  Search modes - Boolean/Phrase | Interface - EBSCOhost  Search Screen - Advanced Search  Database - CINAHL Plus with Full Text |
| S11 | leucotriene | Expanders - Apply related words; Also search within the full text of the articles  Search modes - Boolean/Phrase | Interface - EBSCOhost  Search Screen - Advanced Search  Database - CINAHL Plus with Full Text |
| S10 | (Leukotriene Antagonists) or (MH "Leukotriene Antagonists") or (MH "Leukotrienes") | Expanders - Apply related words; Also search within the full text of the articles  Search modes - Boolean/Phrase | Interface - EBSCOhost  Search Screen - Advanced Search  Database - CINAHL Plus with Full Text |
| S9 | (MH "Administration, Inhalation") or (MH "Nebulizers and Vaporizers") | Expanders - Apply related words; Also search within the full text of the articles  Search modes - Boolean/Phrase | Interface - EBSCOhost  Search Screen - Advanced Search  Database - CINAHL Plus with Full Text |
| S8 | Antiasthmatic Agent* | Expanders - Apply related words; Also search within the full text of the articles  Search modes - Boolean/Phrase | Interface - EBSCOhost  Search Screen - Advanced Search  Database - CINAHL Plus with Full Text |
| S7 | MH asthma or AB asthma or TI asthma | Expanders - Apply related words; Also search within the full text of the articles  Search modes - Boolean/Phrase | Interface - EBSCOhost  Search Screen - Advanced Search  Database - CINAHL Plus with Full Text |
| S6 | S5 or S4 or S3 or S2 or S1 | Expanders - Apply related words; Also search within the full text of the articles  Search modes - Boolean/Phrase | Interface - EBSCOhost  Search Screen - Advanced Search  Database - CINAHL Plus with Full Text |
| S5 | AB Adolescen* or TI Adolescen* | Expanders - Apply related words; Also search within the full text of the articles  Search modes - Boolean/Phrase | Interface - EBSCOhost  Search Screen - Advanced Search  Database - CINAHL Plus with Full Text |
| S4 | AB preschool child* or TI preschool child* | Expanders - Apply related words; Also search within the full text of the articles  Search modes - Boolean/Phrase | Interface - EBSCOhost  Search Screen - Advanced Search  Database - CINAHL Plus with Full Text |
| S3 | AB child* or TI child* | Expanders - Apply related words; Also search within the full text of the articles  Search modes - Boolean/Phrase | Interface - EBSCOhost  Search Screen - Advanced Search  Database - CINAHL Plus with Full Text |
| S2 | AB infant* or TI infant* | Expanders - Apply related words; Also search within the full text of the articles  Search modes - Boolean/Phrase | Interface - EBSCOhost  Search Screen - Advanced Search  Database - CINAHL Plus with Full Text |
| S1 | (MH "Infant") or (MH "Child") or (MH "Child, Preschool") or (MH "Adolescence") | Expanders - Apply related words; Also search within the full text of the articles  Search modes - Boolean/Phrase | Interface - EBSCOhost  Search Screen - Advanced Search  Database - CINAHL Plus with Full Text |

### CENTRAL (Cochrane). [**http://www.mrw.interscience.wiley.com/cochrane/cochrane_clcentral_articles_fs.html**](http://www.mrw.interscience.wiley.com/cochrane/cochrane_clcentral_articles_fs.html). Date last accessed: February 4 2010

| **ID** | **Search** |
| --- | --- |
| #1 | [infant* in Clinical Trials](http://www3.interscience.wiley.com/cochrane/searchHistory?mode=runquery&qnum=1) |
| #2 | [child* in Clinical Trials](http://www3.interscience.wiley.com/cochrane/searchHistory?mode=runquery&qnum=2) |
| #3 | [preschool child* in Clinical Trials](http://www3.interscience.wiley.com/cochrane/searchHistory?mode=runquery&qnum=3) |
| #4 | [adolescen* in Clinical Trials](http://www3.interscience.wiley.com/cochrane/searchHistory?mode=runquery&qnum=4) |
| #5 | [(#1 OR #2 OR #3 OR #4)](http://www3.interscience.wiley.com/cochrane/searchHistory?mode=runquery&qnum=5) |
| #6 | [asthma in Clinical Trials](http://www3.interscience.wiley.com/cochrane/searchHistory?mode=runquery&qnum=6) |
| #7 | [Antiasthmatic Agent* in Clinical Trials](http://www3.interscience.wiley.com/cochrane/searchHistory?mode=runquery&qnum=7) |
| #8 | [Administration Inhalation in Clinical Trials](http://www3.interscience.wiley.com/cochrane/searchHistory?mode=runquery&qnum=8) |
| #9 | [nebulizer* in Clinical Trials](http://www3.interscience.wiley.com/cochrane/searchHistory?mode=runquery&qnum=9) |
| #10 | [Vaporizer* in Clinical Trials](http://www3.interscience.wiley.com/cochrane/searchHistory?mode=runquery&qnum=10) |
| #11 | [Montelukast in Clinical Trials](http://www3.interscience.wiley.com/cochrane/searchHistory?mode=runquery&qnum=11) |
| #12 | [Leukotriene Antagonist* in Clinical Trials](http://www3.interscience.wiley.com/cochrane/searchHistory?mode=runquery&qnum=12) |
| #13 | [Leukotriene* in Clinical Trials](http://www3.interscience.wiley.com/cochrane/searchHistory?mode=runquery&qnum=13) |
| #14 | [Leucotriene* in Clinical Trials](http://www3.interscience.wiley.com/cochrane/searchHistory?mode=runquery&qnum=14) |
| #15 | [pranlukast in Clinical Trials](http://www3.interscience.wiley.com/cochrane/searchHistory?mode=runquery&qnum=15) |
| #16 | [Theophylline in Clinical Trials](http://www3.interscience.wiley.com/cochrane/searchHistory?mode=runquery&qnum=16) |
| #17 | [zafirlukast in Clinical Trials](http://www3.interscience.wiley.com/cochrane/searchHistory?mode=runquery&qnum=17) |
| #18 | [zileuton in Clinical Trials](http://www3.interscience.wiley.com/cochrane/searchHistory?mode=runquery&qnum=18) |
| #19 | [Inhaled Corticosteroid* in Clinical Trials](http://www3.interscience.wiley.com/cochrane/searchHistory?mode=runquery&qnum=19) |
| #20 | [Budesonide in Clinical Trials](http://www3.interscience.wiley.com/cochrane/searchHistory?mode=runquery&qnum=20) |
| #21 | [Fluticasone in Clinical Trials](http://www3.interscience.wiley.com/cochrane/searchHistory?mode=runquery&qnum=21) |
| #22 | [ciclesonide in Clinical Trials](http://www3.interscience.wiley.com/cochrane/searchHistory?mode=runquery&qnum=22) |
| #23 | [flunisolide in Clinical Trials](http://www3.interscience.wiley.com/cochrane/searchHistory?mode=runquery&qnum=23) |
| #24 | [mometasone furoate in Clinical Trials](http://www3.interscience.wiley.com/cochrane/searchHistory?mode=runquery&qnum=24) |
| #25 | [Triamcinolone Acetonide in Clinical Trials](http://www3.interscience.wiley.com/cochrane/searchHistory?mode=runquery&qnum=25) |
| #26 | [dry powder inhaler* in Clinical Trials](http://www3.interscience.wiley.com/cochrane/searchHistory?mode=runquery&qnum=26) |
| #27 | [Metered Dose Inhaler* in Clinical Trials](http://www3.interscience.wiley.com/cochrane/searchHistory?mode=runquery&qnum=27) |
| #28 | [Sustained-release tablet* in Clinical Trials](http://www3.interscience.wiley.com/cochrane/searchHistory?mode=runquery&qnum=28) |
| #29 | [Beclomethasone in Clinical Trials](http://www3.interscience.wiley.com/cochrane/searchHistory?mode=runquery&qnum=29) |
| #30 | [Adrenergic Beta-Agonist* in Clinical Trials](http://www3.interscience.wiley.com/cochrane/searchHistory?mode=runquery&qnum=30) |
| #31 | [Long-acting beta-2-agonist* in Clinical Trials](http://www3.interscience.wiley.com/cochrane/searchHistory?mode=runquery&qnum=31) |
| #32 | [Long-acting beta agonist* in Clinical Trials](http://www3.interscience.wiley.com/cochrane/searchHistory?mode=runquery&qnum=32) |
| #33 | [Long-acting beta2-agonist* in Clinical Trials](http://www3.interscience.wiley.com/cochrane/searchHistory?mode=runquery&qnum=33) |
| #34 | [Sympathomimetics in Clinical Trials](http://www3.interscience.wiley.com/cochrane/searchHistory?mode=runquery&qnum=34) |
| #35 | [LABA in Clinical Trials](http://www3.interscience.wiley.com/cochrane/searchHistory?mode=runquery&qnum=35) |
| #36 | [formoterol in Clinical Trials](http://www3.interscience.wiley.com/cochrane/searchHistory?mode=runquery&qnum=36) |
| #37 | [Salmeterol in Clinical Trials](http://www3.interscience.wiley.com/cochrane/searchHistory?mode=runquery&qnum=37) |
| #38 | [albuterol in Clinical Trials](http://www3.interscience.wiley.com/cochrane/searchHistory?mode=runquery&qnum=38) |
| #39 | [Salbutamol in Clinical Trials](http://www3.interscience.wiley.com/cochrane/searchHistory?mode=runquery&qnum=39) |
| #40 | [Tablet* in Clinical Trials](http://www3.interscience.wiley.com/cochrane/searchHistory?mode=runquery&qnum=40) |
| #41 | [Terbutaline in Clinical Trials](http://www3.interscience.wiley.com/cochrane/searchHistory?mode=runquery&qnum=41) |
| #42 | [aminophylline in Clinical Trials](http://www3.interscience.wiley.com/cochrane/searchHistory?mode=runquery&qnum=42) |
| #43 | [theophyllamine in Clinical Trials](http://www3.interscience.wiley.com/cochrane/searchHistory?mode=runquery&qnum=43) |
| #44 | [ICS in Clinical Trials](http://www3.interscience.wiley.com/cochrane/searchHistory?mode=runquery&qnum=44) |
| #45 | [beta adrenergic receptor stimulating agent* in Clinical Trials](http://www3.interscience.wiley.com/cochrane/searchHistory?mode=runquery&qnum=45) |
| #46 | [(#7 OR #8 OR #9 OR #10 OR #11 OR #12 OR #13 OR #14 OR #15 OR #16 OR #17 OR #18 OR #19 OR #20 OR #21 OR #22 OR #23 OR #24 OR #25 OR #26 OR #27 OR #28 OR #29 OR #30 OR #31 OR #32 OR #33 OR #34 OR #35 OR #36 OR #37 OR #38 OR #39 OR #40 OR #41 OR #42 OR #43 OR #44 OR #45)](http://www3.interscience.wiley.com/cochrane/searchHistory?mode=runquery&qnum=46) |
| #47 | [(#5 AND #6 AND #46)](http://www3.interscience.wiley.com/cochrane/searchHistory?mode=runquery&qnum=47) |

WHO search portal. Search portal for ongoing trials. <http://apps.who.int/trialsearch/>. Date last accessed: February 5 2010.

**Search method 1**:

Clinical trials in Children: Advanced search: asthma

**Search method 2**: advanced search, Search ‘clinical trials in children’; at condition ‘asthma’ and at Intervention ‘nebulizer OR vaporizer OR montelukast OR leukotrine

pranlukast OR theophylline OR zafirlukast OR zileuton OR budesonide OR inhaled corticosteroids

fluticasone OR ciclesonide OR flunisolide OR mometasone furoate OR Triamcinolone Acetonide

dry powder inhaler OR Metered Dose Inhaler OR beclomethasone OR long-acting beta agonist

formoterol OR Salmeterol OR albuterol OR Salbutamol OR Terbutaline OR aminophylline OR theophyllamine OR ICS’
