# Supplementary material for: A systematic review with attempted network meta-analysis of asthma therapy recommended for five to eighteen year olds in GINA steps three and four
Source: BMC Pulm Med. 2012 Oct 15;12:63. doi: 10.1186/1471-2466-12-63 (PMC3582530; doi:10.1186/1471-2466-12-63)
Supplement: Additional file 2 — Appendix 2. Medication options GINA step 3&4 for 5-18 year olds. [file 1471-2466-12-63-S2.doc]

**Appendix 2. Medication options GINA step 3&4 for 5-18 year olds.**

| **GINA 3A** | **Combination low dose ICS + Long acting β2-agonist** | | |
| --- | --- | --- | --- |
|  | **DPI** | **OR** | **MDI** |
| Fluticasone/salmeterol | 100-200μg/50μg bid |  | 50-250 μg/25μg bid |
| Budesonide/formoterol | 100 or 200μg/6μg bid |  | 80 or 160μg/25μg bid |

| **GINA 3B** | **Medium or High dose ICS** | | |
| --- | --- | --- | --- |
| **Medium dose ICS** | **Dose (µg)** | **High dose ICS** | **Dose (µg)** |
| Beclomethasone | >200-400 | Beclomethasone | >400 |
| Budesonide | >200-400 | Budesonide | >400 |
| Budesonide Neb | >500-1000 | Budesonide Neb | >1000 |
| Ciclesonide | >160-320 | Ciclesonide | >320 |
| Flunisolide | >750-1250 | Flunisolide | >1250 |
| Fluticasone | >200-500 | Fluticasone | >500 |
| Mometasone (furoate) | >200-400 | Mometasone (furoate) | >400 |
| Triamcinolone (acetonide) | >800-1200 | Triamcinolone (acetonide) | >1200 |

| **GINA 3C** | **Comination Low dose ICS + Antileukotrienes** | | |
| --- | --- | --- | --- |
| **Lowe dose ICS** | **Dose (µg)** | **Antileukotrienes** | **Dose mg** |
| Beclomethasone | 100-200 | Montelukast | 5 qhs (6-14 yr) |
| Budesonide | 100-200 |  | 10 qhs (adolescents) |
| Budesonide Neb | 250-500 | Pranlukast | 450 bid (adolescents) |
| Ciclesonide | 80-160 | Zafirlukast | 10 bid (7-11 yr) |
| Flunisolide | 500-750 |  | 20 bid (adolescents) |
| Fluticasone | 100-200 | Zileuton | 600 qid (adolescents) |
| Mometasone (furoate) | 100-200 |  | |
| Triamcinolone (acetonide) | 400-800 |  | |

| **GINA 3D** | **Combintaion Low dose ICS + SR theophylline** | | |
| --- | --- | --- | --- |
| **Lowe dose ICS** | **Dose (µg)** | **Sustained released theophylline** | **Dose** |
| Beclomethasone | 100-200 | Amniophylline | starting dose 10 mg/kg/day; max 800 mg in 1-2 doses |
| Budesonide | 100-200 | Methylxanthine |
| Budesonide Neb | 250-500 | Xanthine |
| Ciclesonide | 80-160 |  |
| Flunisolide | 500-750 |  | |
| Fluticasone | 100-200 |  | |
| Mometasone (furoate) | 100-200 |  | |
| Triamcinolone (acetonide) | 400-800 |  | |

| **GINA 4A** | **Combination Medium or High dose ICS + Long acting β2-agonist** |
| --- | --- |

| **GINA 4B** | **Add Antileukotrienes to GINA step 3** | |
| --- | --- | --- |
| **Antileukotrienes** | **Dose mg** | |
| Montelukast | 5 qhs (6-14 yr) | 10 qhs (adolescents) |
| Pranlukast | 450 bid (adolescents) | |
| Zafirlukast | 10 bid (7-11 yr) | 20 bid (adolescents) |
| Zileuton | 600 qid (adolescents) | |

| **GINA 4C** | **Add SR theophylline to GINA step 3** | |
| --- | --- | --- |
| **SR theophylline** | | **Dose** |
| Amniophylline | | starting dose 10 mg/kg/day usual 800 mg max in 1-2 doses |
| Methylxanthine | |
| Xanthine | |

Abbreviations:

ICS: inhaled glucocorticosteroids

DPI: dry powder inhaler

MDI: Metered Dose Inhaler

bid: twice a day

qid: four times a day

qhs: at every bedtime

SR: sustained release
