# Supplementary material for: A systematic review with attempted network meta-analysis of asthma therapy recommended for five to eighteen year olds in GINA steps three and four
Source: BMC Pulm Med. 2012 Oct 15;12:63. doi: 10.1186/1471-2466-12-63 (PMC3582530; doi:10.1186/1471-2466-12-63)
Supplement: Additional file 3 — Appendix 3. Statistical Analysis – The modeling used for FEV1%pred. [file 1471-2466-12-63-S3.doc]

**Appendix 3. Statistical Analysis – The modeling used for FEV_1_%_pred_**

Given a mean age and its range or standard deviation (SD), 1000 patient ages may be simulated. Using such simulation, we created age-, height-, and sex-specific FEV_1_ values for 1000 virtual children from a general population (the denominator of formula 1 in figure 2) in three steps. First, we used the reported summary statistics for each trial arm to draw 1000 simulated children’s age- (assuming a uniform distribution), and sex combinations. Height was calculated using the World Health Organization data.[19] Second, once we had 1000 simulated children’s age, height, and sex value combinations, we calculated the corresponding FEV_1_ values using the formulas (see table 5).[20-23]

Thus, we obtained 1000 values of the denominator of formula 1 in the figure 2. The numerator in the formula is necessary to calculate FEV_1_%_pred_, but an additional assumption was needed, because the publications did not report age-, height, and sex-specific data. We assumed that the difference between an asthmatic child’s age-, height-, and sex-specific FEV_1_ and the corresponding FEV_1_ from a child from the general population (which we had simulated, N=1000) was equal to the difference between the mean FEV_1_, as reported in each particular trial publication (on asthmatic children) and the mean FEV_1_ from the general population (which we had simulated, N=1000). This assumption allows the calculation of an asthmatic child’s age-, height-, and sex-specific FEV_1_ for each of the 1000 simulated children by solving for FEV_1_(a,h,s)_asthmatic_ (see formula 2 in the figure 3). Thus, the numerator in formula 1 is complete and a FEV_1_%_pred_ may be calculated for each (simulated) child. In a final step, for each trial-arm, a mean FEV_1_%_pred_ and a corresponding SD may be calculated from the simulated data, to be used for meta-analysis.

**Table 5. Published FEV_1_ regression formulas.**

| **Reference** | **Age (years)** | **Male/**  **Female** | **Function (FEV_1_)** |
| --- | --- | --- | --- |
| Polgar 1971[21] | 6-18 | Male | 0.001 * 10^(-2.6778 + 2.7986 * log10 (Heightcm))^ |
| Polgar 1971 | 6-18 | Female | 0.001 * 10^(-3.1113 + 3.00* log10 (Heightcm))^ |
| Hankinson 1999[20] | <20 | Male | -0.7453 - 0.04106 * Age + 0.004477 * Age² + 0.00014098 * (Height_cm_)² |
| Hankinson 1999 | <18 | Female | -0.8710 + 0.06537 * Age + 0.00011496 * Heightcm² |
| Quanjer 1995[22] | 6-21 | Male | exp((1.2669 + 0.0174 * Age) * Height_cm_/100.0 - 1.2933) |
| Quanjer 1995 | 6-18 | Female | exp((1.5016 + 0.0119*Age) * Height_cm_/100.0 - 1.5974) |
| Zapletal 1971[23] | 6-17 | Male | 0.001 * 10^(-2.86521 + 2.87294*log10(Heightcm))^; (log) RSD = 0.0312 |
| Zapletal 1971 | 6-17 | Female | 0.001 * 10^(-2.60565 + 2.74136*log10(Heightcm))^; (log) RSD = 0.0483 |

The RSD is the residual standard deviation, that is, the standard deviation about the predicted value after allowing for age and height.

**Figure 3**

FEV_1_ (a,h,s) _asthmatic patient_

FEV_1_ (a,h,s) _general population_

FEV_1_%_pred_ =

FEV_1_ (a,h,s) _asthmatic_ - FEV_1_ (a,h,s) _general population_ = mean (FEV1study_i) – mean (FEV_1 general population_)

[Formula 1]

[Formula 2]

**Title:**

Formula’s for calculating FEV_1_-values

**Abbreviations:**

FEV_1_ = forces expiratory volume in one second; a = age; h =height; s = sex

**Legend:**

Formula 1. Formula to calculate the percentage of predicted, per individual.

Formula 2. Assumption that the difference between an asthmatic child’s age-, height-, and sex-specific FEV_1_ and the corresponding FEV_1_ from a child from the general population is equal to the difference between the mean FEV_1_, as reported in each trial and the mean FEV_1_ from the general population.

Reference List

19. WHO. World Health Organisation. 7-4-0010.

Online Source

20. Hankinson JL, Odencrantz JR, Fedan KB: **Spirometric reference values from a sample of the general U.S. population.** *Am J Respir Crit Care Med* 1999, **159:** 179-187.

21. Polgar G: **Pulmonary function tests in children.** *J Pediatr* 1979, **95:** 168-170.

22. Quanjer PH, Borsboom GJ, Brunekreef B, Zach M, Forche G, Cotes JE *et al*.: **Spirometric reference values for white European children and adolescents: Polgar revisited.** *Pediatr Pulmonol* 1995, **19:** 135-142.

23. Zapletal A, Paul T, Samanek M: **Die Bedeutung heutiger Methoden der Lungenfunktionsdiagnostik zur Feststellung einer Obstruktion det Atemwege bei Kindern und Jugendlichen.** *Z Erkr Atmungsorgane* 1977, **149:** 343-371.
