# Supplementary material for: A systematic review with attempted network meta-analysis of asthma therapy recommended for five to eighteen year olds in GINA steps three and four
Source: BMC Pulm Med. 2012 Oct 15;12:63. doi: 10.1186/1471-2466-12-63 (PMC3582530; doi:10.1186/1471-2466-12-63)
Supplement: Additional file 4 — Appendix 4. Study characteristics. [file 1471-2466-12-63-S4.doc]

**Appendix 4. Study characteristics**

| **ID** | **Author**  **Year of Publication** | **GINA**  **ref** | **Population**  **(Number of participants & Age)** | **Drug treatment per trial arm** | **Duration (weeks)** | **Outcomes** |
| --- | --- | --- | --- | --- | --- | --- |
| 1 | Bennati  1989 | - | N=30  Mean age 9.6  Range age 6-14 | Group 1: beclomethasone 300 pMDI  Group 2: beclomethasone 300 pMDI  Group 3: placebo | 4 | Methacholine PC_20_- FEV_1_ |
| 2 | Verini  2007 | - | N= 24  Mean age 9.5  Range age 6-13 | Group 1: fluticasone 200 DPI + salmeterol 100  Group 2: fluticasone 200 DPI + montelukast 5 | 4 | PEF%  FEV_1_%_pred_  FVC%  FEF_25-75_  eNO |
| 3 | Miraglia del Giudice  2007 | - | N= 48  Mean age  Range age 7-11 | Group 1: budesonide 400 DPI  Group 2: budesonide 400 DPI/formoterol 18  Group 3: budesonide 400 DPI/montelukast 5  Group 4: budesonide 800 DPI | 4  (group 3: 8) | FEV_1_%_pred_  eNO |
| 4 | Kondo  2006 | - | N= 75  Mean age 9.4 & 8.8  Range age 6-14 | Group 1: beclomethasone 100-400 pMDI or fluticasone 100-200 + montelukast 5  Group 2: beclomethasone 100-400 or fluticasone 100-200 + theophylline 10-16 mg/kg/d or 200-400 mg/d | 4 | PEF (l/min)  PEF%  Mild attacks  β_2_-agonist use |
| 5 | Boner  1991 | - | N= 20  Mean age 8.8 & 9.7  Range age | Group 1: beclomethasone 300 pMDI  Group 2: placebo | 8 | FEV_1_%_pred_  Methacholine PC_20_- FEV_1_ |
| 6 | Stelmach  2006 | - | N= 85  Mean age 11.0 & 8.79 & 11.4  Range age 6-18 | Group 1: budesonide 200 DPI + montelukast 5/10  Group 2: budesonide 200 DPI + formoterol 9  Group 3: placebo | 8 | FEV_1_%_pred_  FEF_25-75_ |
| 7 | Meltzer  1985 | - | N= 52  Mean age 10.3 & 9.5  Range age 6-12 | Group 1: beclomethasone 336 pMDI  Group 2: placebo | 10 | FEV_1_%_pred_  FVC%  FEF_25-75_  Symptoms |
| 8 | Piacentini  1990 | - | N= 20  Mean age 10.15  Range age 8-13 | Group 1: flunisolide 1000 pMDI  Group 2: placebo | 8 | PEF(l/min)  FEV_1_ (l)  FVC(l)  FEF_25-75_ (1/s)  Methacholine PC_20_- FEV_1_ (mg/ml)  Symptoms |
| 9 | Jat  2006 | 201 | N= 63  Mean age 10.13 (SD 2.67) & 9.34 (SD 2.67)  Range age 6-14 | Group 1: budesonide 200 DPI + montelukast 5  Group 2: budesonide 400 DPI | 12 | PEF(l/min)  PEF%  FEV_1_%_pred_  Symptoms  Exacerbations (nr) |
| 10 | Shapiro  1998 | 128 | N= 131  Mean age 6.85 (SD 1.40) & 6.80 (SD 1.26) & 6.73 (SD 1.51)  Range age 4-9 | Group 1: budesonide 1000 pMDI  Group 2: budesonide 2000 pMDI  Group 3: placebo | 12 | PEF (l/min) morning & evening  FEV_1_ (l)  FEV_1_%_pred_  Symptoms  β_2_-agonist use |
| 11 | Shapiro  2001 | - | N= 184  Mean age 12.10 (SD 2.8 & 3.0)  Range age 6-18 | Group 1: budesonide 400 DPI  Group 2: placebo | 12 | PEF% morning & evening  FEV_1_ (l)  FEV_1_%_pred_  FVC (l)  FEF_25-75_ (1/s)  Symptoms  β_2_-agonist use |
| 12 | Shapiro  1998 | - | N= 302  Mean age 12.00 & 12.10 & 11.80  Range age 6-18 | Group 1: placebo  Group 2: budesonide 400 DPI  Group 3: budesonide 800 DPI | 12 | PEF% morning  FEV_1_%_pred_  Symptoms  β_2_-agonist use |
| 13 | Tal  2002 | - | N= 286  Mean age 11.0  Range age 4-17 | Group 1: budesonide 320 /formoterol 18 DPI  Group 2: budesonide 400 DPI | 12 | PEF% morning & evening  PEF(l/min) morning & evening  FEV_1_ (l)  FEV_1_%_pred_  Symptoms  β_2_-agonist use  Nocturnal awakenings%  Symptom-free days% |
| 14 | Morice  2007 | - | N= 622  Mean age 9.0 & 8.0 & 8.0  Range age 6-11 | Group 1: budesonide 400 DPI  Group 2: budesonide 320/formoterol 18 pMDI  Group 3: budesonide 320 /formoterol 18 DPI | 12 | PEF(l/min)  PEF%  FEV_1_ (l)  FEV_1_%_pred_  β_2_-agonist use  Symptoms  Nocturnal awakenings%  Symptom-free days%  asthma control days%  PAQLQ |
| 15 | Pohunek  2006 | - | N= 630  Mean age 8.2 & 8.1 & 8.1  Range age 4-11 | Group 1: budesonide 400 DPI  Group 2: budesonide 400 + formoterol 18 DPI  Group 3: budesonide 320 /formoterol 18 DPI | 12 | PEF (l/min) morning & evening  FEV_1_ (l)  FEV_1_%_pred_  β_2_-agonist use  Symptoms  Nocturnal awakenings%  Symptom-free days%  Asthma control days% (reliever free days)  PAQLQ  Adverse events |
| 16 | Meijer  1995 | 168 | N= 40  Mean age 11.4 (SD 2.4) & 11.4 (SD 2.8)  Range age 7-15 | Group 1: beclomethasone 400/800 DPI + salmeterol 100  Group 2: beclomethasone 400/800 DPI | 16 | PEF (l/min) morning & evening  PEF % morning & evening  FEV_1_ (l)  FEV_1_%_pred_  FVC (l/min)  FVC %  FEV_1_/FVC  Methacholine PC_20_- FEV_1_ (mg/ml)  Symptoms |
| 17 | Gappa  2009 | - | N=283  Mean age 9.6 (SD 3.1) & 9.4 (SD 3.1)  Range age 4-16 | Group 1: fluticasone 200 + salmeterol 100 DPI  Group 2: fluticasone 400 DPI | 8 | PEF (l/min) morning & evening  PEF (%) morning & evening  FEV_1_%_pred_  FVC %  Symptoms  reliever free days%  β_2_-agonist use free days% |
| 18 | Estelle R. Simons  1997 | - | N= 161  Mean age 9.6 (SD 2.6) & 9.5 (SD 2.5)  Range age 6-14 | Group 1: beclomethasone 400 DPI  Group 2: placebo | 56 | PEF (l/min) morning  FEV_1_ (l)  FEV_1_%_pred_  FVC (l)  FEF_25-75_ (1/s)  Methacholine PC_20_- FEV_1_ (mg/ml)  Symptoms |
| 19 | Bisgaard  2006 | 347 | N= 341  Mean age 8  Range age 4-11 | Group 1: budesonide 320 DPI  Group 2: budesonide 80 /formoterol 4.5 fixed (DPI)  Group 3: budesonide 80 /formoterol 4.5 SMART (DPI) | 52 | PEF (l/min) morning & evening  FEV_1_ (l)  FEV_1_%_pred_  Symptoms  Nocturnal awakenings  β_2_-agonist use  Reliever free days%  Symptom-free days%  Asthma control days% (symptom free days(%) & no β_2_-agonist use) |
| 20 | Ilowite  2004 | - | N= 67  Mean age 15.8 (SD 0.9) & 15.7 (SD 0.8)  Range age 14-17 | Group 1: fluticasone 220 + montelukast 10 pMDI  Group 2: fluticasone 220 + salmeterol 84 pMDI | 48 | PEF (l/min) morning & evening  FEV_1_%_pred_  Daytime symptoms  Symptom-free days  β_2_-agonist use |
| 21 | Verberne  1998 | 151 | N= 177  Mean age 10.8 (SD 2.5)%11.4(SD 2.9)&11.1(SD2.7)  Range age 6-16 | Group 1: beclomethasone 400 DPI+Salmeterol 100 µg  Group 2: beclomethasone 800 DPI  Group 3: beclomethasone 400 DPI | 54 | FEV_1_%_pred_  PEF (l/min) morning & evening  Symptoms  β_2_-agonist use  Methacholine PD_20_ |
| 22 | Trial  2008 | - | N=186  Mean age 9.0 (SD 1.624)  Range age 6-11 | Group 1: budesonide 320 µg pMDI + formoterol 9 µg  Group 2: budesonide 400 TBH | 26 | FEV_1_ (l)  FEV_1_%_pred_  PAQLQ |
| 23 | Blic  2009 | - | N=303  Mean age 8.1 & 8.0  Range age 4-11 | Group 1: fluticasone 400 DPI  Group 2: fluticasone 200 + salmeterol 100 DPI | 12 | PEF (l/min) morning  FEV_1_ (l)  Symptoms  Nocturnal awakenings  β_2_-agonist use  reliever free days%  MEF_50_ (l/s) |

**Abbreviations:**

**Medication**

pMDI = pressurized metered-dose inhaler

DPI = dry powder inhaler

TBH = Turbuhaler

SMART = Symbicort maintenance and relief therapy
