# Supplementary material for: A systematic review with attempted network meta-analysis of asthma therapy recommended for five to eighteen year olds in GINA steps three and four
Source: BMC Pulm Med. 2012 Oct 15;12:63. doi: 10.1186/1471-2466-12-63 (PMC3582530; doi:10.1186/1471-2466-12-63)
Supplement: Additional file 5 — Appendix 5. Measured and reported Asthma Symptoms and Inhaled β2-agonist use per trial. [file 1471-2466-12-63-S5.doc]

**Appendix 5. Measured and reported Asthma Symptoms and Inhaled β2-agonist use per trial.**

| Study ID | Scored on symptoms | Method | Scale | Reported | Items scored on |
| --- | --- | --- | --- | --- | --- |
| 1 | Y | Daily diary |  | - No results for symptoms are reported.  - Average frequency of inhaled β2-agonist use in the groups.  [see quote] | Cough, wheezing in the night and day;  Medication-use (co-medication and inhaled β2-agonist use). |
| 2 | N |  |  |  |  |
| 3 | N |  |  |  |  |
| 4 | Y | Daily diary |  | - Mild attacks^a^ (mean times/week+SE)  - Inhaled β2-agonist use (mean times/week+SE)(+p-values of mean changes) | Asthma-attacks;  Cough;  Activities;  Nighttime sleep;  Compliance;  Inhaled β2-agonist use |
| 5 | N |  |  |  |  |
| 6 | N |  |  |  |  |
| 7 | Y | Daily diary | 0-4^b^ | - Mean score + SE on a 0-4 scale per item (wheezing, breathlessness, chest tightness, cough)  - overall statement about Inhaled β2-agonist use  [see quote] | Wheezing, breathlessness, chest tightness, cough;  Inhaled β2-agonist use |
| 8 | Y | Daily diary |  | - overall statement about symptoms + p-value  - overall statement about Inhaled β2-agonist use  [see quote] | Cough, wheezing, dyspnea, side effects. |
| 9 | Y | Daily diary | 0-6 (daily; 0-42 weekly) | Weekly symptom score; results are shown in a figure. (Author supplied us the mean + SD per group per week)  [see quote] | 6 items: Cough, wheezing, difficulty in breathing, missing of school, exercise intolerance, inhaled β2-agonist use. (Absent=0, present=1.) |
| 10 | Y | Daily diary | 0-3 (day and night)^c^ | Mean changes over 12 weeks of:  - Nighttime asthma symptom score  - Daytime asthma symptom score  In case of significant difference vs placebo, p<0.05 or p<0.01 is given. | Inference of daily activities and nighttime sleep because of asthma or cough;  Inhaled β2-agonist use |
| 11 | Y | Daily electronic diariy | 0-3 (day and night)^c^ | - Mean daytime score + SD  - Mean nighttime score + SD  - β2-agonist use (Mean no. of puff/day + SD) | Inference of daily activities and nighttime sleep because of asthma or cough;  Inhaled β2-agonist use |
| 12 | Y | Daily diary | 0-3 (day and night)^c^ | Symptoms:  - p-values of placebo vs treated groups of mean changes;  - p-value of dose response  Inhaled β2-agonist use:  - % of decrease in the number of bronchodilator inhalations used per day per group  - p-value vs placebo & dose response  [see quote] | Inference of daily activities and nighttime sleep because of asthma or cough;  Inhaled β2-agonist use |
| 13 | Y | Daily diary | 0-3 | - Mean symptom score (0-6)  - % of symptom-free days  - Mean % of nighttime awakenings  - Inhaled β2-agonist use (Mean no. of puff/day + SD)  + between-group difference (95% CI) | Severity of daytime & nighttime asthma symptoms^d^;  Nighttime awakenings;  Inhaled β2-agonist use. |
| 14 | Y | Daily diary | 0-6 | Mean change of:  - symptom score  - % of nighttime awakenings  - % of symptom-free days  - % of asthma-control days  - inhaled β2-agonist use (puff/day)  In case of significant difference between groups, p-value + 95%CI is given. | Symptoms;  Symptom-free days^e^;  Asthma-control days^f^ |
| 15 | Y | Daily diary | 0-6 | - Mean symptom score (0-6)  - % of symptom-free days  - % of nighttime awakenings  - Inhaled β2-agonist use (Mean no. of puff/day)  - % of inhaled β2-agonist-free days | Symptoms (sum of day&night symptom scores);  Nighttime awakenings due to astma symptoms;  Symptom-free days;  Inhaled β2-agonist use |
| 16 | Y | Daily diary |  | - Number of children with symptoms during baseline or treatment period.  - Number of children using inhaled β2-agonist during baseline or treatment period. | For day and night: wheeze, dyspnea, cough, phlegm;  Inhaled β2-agonist use |
| 17 | Y | Daily diary | 0-9 | p-values of the differences of:  - daytime symptom scores  - nighttime symptom scores  - inhaled β2-agonist use  [see quote] | Severity of symptoms, measured by inference of daily activities and nighttime sleep because of asthma symptoms^g^;  Inhaled β2-agonist use |
| 18 | Y | Daily diary |  | Over 12 months:  - % of days&nights inhaled β2-agonist-free days  - % of children inhaled β2-agonist not required  - % of children no school missed because of asthma  - % of days activities affected by asthma  - % of nighttimes awaking | Inhaled β2-agonist use for breakthrough wheezing, symptoms of asthma, absences from school night waking. |
| 19 | Y | Daily diary | 0-6 | - Mean symptom score (0-6)  - % of symptom-free days^h^  - % of nighttime awakenings  - Daytime inhaled β2-agonist use (Mean no. of puff/day)  - Nighttime inhaled β2-agonist use (Mean no. of puff/day)  - % of inhaled β2-agonist-free days  - % of Asthma-control days^i^ | Severity of symptoms, measured by inference of daily activities and nighttime sleep because of asthma symptoms; Nighttime awakenings^j^;  Inhaled β2-agonist use. |
| 20 | Y | Daily diary | 0-6 | - Mean daytime score +SD  - Mean symptom-free days (days/week)+ SD  - Inhaled β2-agonist use (Mean no. of puff/day + SD) | Daytime symptoms scores; Asthma attacks;  Inhaled β2-agonist use. |
| 21 | Y | Daily diary | 0-3 | - % of children reporting no symptoms  - median number of inhaled β2-agonist use per day  In case of significant difference between groups, p-value is given.  [see quote] | For morning and evening:  Dyspnea, wheeze, cough;  Inhaled β2-agonist use. |
| 22 | N |  |  |  |  |
| 23 | Y | Daily diary | 0-5 | - % of children with  0-<25%  25-<50%  50-<75%  75-<100%  100%  of symptom-free days during study.  - Mean nr. Of nighttime awakenings + SE + 95%CI of between group difference, p-value  - % of children with  0-<25%  25-<50%  50-<75%  75-<100%  100%  of inhaled β2-agonist-free days. | Symptoms^k^;  Nighttime awakenings;  Inhaled β2-agonist use. |

^a^an episode of mild wheezing occasionally associated with mild intercostal or tracheosternal retractions.

^b^0 for no symptoms and 4 for unbearable symptoms

^c^Day: 0= none; 1= mild, symptoms noticeable, but not enough to cause trouble with daily routine and activities; 2= moderate, symptoms noticed often, causing some inference with daily routine and activities; 3= severe, symptoms continuous or present most of the day, severely restricting daily routine and activities.

Night: 0= none; 1= mild awoke once because of asthma or cough; 2= moderate, awoke more than once because of asthma or cough; 3= severe, awake most of the night because of asthma or cough.

^d^0= no symptoms, 3= severe symptoms

^e^Symptom-free day= a night and day without asthma-symptoms and no nighttime awakenings caused by asthma

^f^Asthma-control day= a night and day without asthma-symptoms or reliever medication use and no nighttime awakenings caused by asthma

^g^Day: 0= none, 1= 1 short period, 2= ≥2 short periods, 3= most of the day (activity not affected), 4= most of the day (activity reduced), 5= severe, unable to go to school.

^h^Symptom-free day= a day and night with no asthma symptoms or awakenings due to asthma symptoms.

^i^Asthma-control day= a symptom-free day with no reliever medication use.

^j^Night: 0= none, 1= wakened once, 2= wakened ≥2 times, 3= awake most of the night, 4= severe (unable to sleep); Total: 0-9

^k^ Based on a six-point scale from 0 (no symptoms) to 5 (symptoms so severe that the subject could not attend school or perform normal activities).

**Quotes:**

Study 1:

“The use of comedication was practically absent in the first group, occasional (once or twice a week) in the second group, and of course, more frequent (at least once a day) in the third group.”

Study 7:

“Placebo-treated patients needed more concomitant medication for asthma control than did BDP-treated patients.”

Study 8:

“Symptom evaluation showed a significant decrease in the number of diurnal asthmatic episodes at the end of the study period for the flunisolide group(p<0.01). No change was observed in the placebo group. Patients’ other symptoms as well drug consumption remained unchanged”

Study 9:

“Asthma symptom score showed a significant decline by approximately 4 points in both groups after the first week of intervention (p<0.05). Thereafter remained almost constant. There was no significant difference between groups at any of the follow-up visits.”

Study 12:

“The mean changes from baseline between the placebo group and each of the budesonide groups were significant (p<0.001) for the period from baseline to week 12. A dose respons was observed for the decrease in daytime symptoms, which was significantly greater after treatment with 400 µg of budesonide given twice daily that after treatment with 100 µg of budesonide given twice daily(p=0.041). No significant diference was observed between the group treated twice daily 100 µg of budesonide and the group given 400 µg of budesonide given twice daily for the nighttime asthma symptoms.”

“A 26% increase in the number of bronchodilator inhalations used per day was observed in the placebo-treated group, whereas a mean decrease of 24%, 30% and 40% per day was seen in the groups receiving 100, 200 and 400 µg of budesonide twice daily, respectively. The difference between each budesonide group and placebo was statistically significant (p<0.001). A dose-response relationship in favour of the highest compared with the lowest dose of budesonide was observed (p=0.036).”

Study 17:

“There were no differences between treatment groups for improvements in daytime symptom scores (p=0.729), nighttime scores (p=0.34), or % of days with symptom scores <2 (p=0.107). Nighttime scores were equally reduced in both groups (p= 0.958). Similarly, the need for rescue medication was similar in the 2 groups during the treatment phase for daytime (p=0.181) and nighttima (p=0.59) albuterol use and for ability to completely withfraw albuterol, day (p=0.134) or night (p=0.507).”

Study 21:

“The percentage of children reporting no symptoms during the 2-wk diary card periods increased from 3%, 13%, and 11% for the BDP4001salm, BDP800, and BDP400 groups, respectively in the run-in period to 34%, 39%, and 35% after 1 yr of treatment. At no time point were there statistically significant differences in symptom scores between the groups.“

“The median number of additional salbutamol inhalations per day during the whole treatment period, as counted from the used blisters, was 0.19, 0.33, and 0.15 for the BDP4001salm, BDP800, and BDP400 groups, respectively. The difference between the rates in the BDP800 and BDP400 group was of borderline statistical significance (p= 0.06).”
